# Tilting and rotational motions of silver halide crystal with diffracted X-ray blinking

**DOI:** 10.1038/s41598-021-83320-y

**Published:** 2021-03-05

**Authors:** Masahiro Kuramochi, Hiroki Omata, Masaki Ishihara, Sander Ø. Hanslin, Masaichiro Mizumaki, Naomi Kawamura, Hitoshi Osawa, Motohiro Suzuki, Kazuhiro Mio, Hiroshi Sekiguchi, Yuji C. Sasaki

**Affiliations:** 1 Graduate School of Frontier Sciences, The University of Tokyo, Kashiwa, 277-8561 Japan; 2 AIST-UTokyo Advanced Operando-Measurement Technology Open Innovation Laboratory (OPERANDO-OIL), National Institute of Advanced Industrial Science and Technology (AIST), Kashiwa, 277-0882 Japan; 3 Center for Synchrotron Radiation Research, Japan Synchrotron Radiation Research Institute, 1-1-1, Kouto, Sayo-cho, Sayo-gun, Hyogo 679-5198 Japan

**Keywords:** Techniques and instrumentation, Nanometrology

## Abstract

The dynamic properties of crystalline materials are important for understanding their local environment or individual single-grain motions. A new time-resolved observation method is required for use in many fields of investigation. Here, we developed in situ diffracted X-ray blinking to monitor high-resolution diffraction patterns from single-crystal grains with a 50 ms time resolution. The diffraction spots of single grains of silver halides and silver moved in the θ and χ directions during the photolysis chemical reaction. The movements of the spots represent tilting and rotational motions. The time trajectory of the diffraction intensity reflecting those motions was analysed by using single-pixel autocorrelation function (sp-ACF). Single-pixel ACF analysis revealed significant differences in the distributions of the ACF decay constants between silver halides, suggesting that the motions of single grains are different between them. The rotational diffusion coefficients for silver halides were estimated to be accurate at the level of approximately 0.1 to 0.3 pm^2^/s. Furthermore, newly formed silver grains on silver halides correlated with their ACF decay constants. Our high-resolution atomic scale measurement—sp-ACF analysis of diffraction patterns of individual grains—is useful for evaluating physical properties that are broadly applicable in physics, chemistry, and materials science.

## Introduction

**Figure 1:**
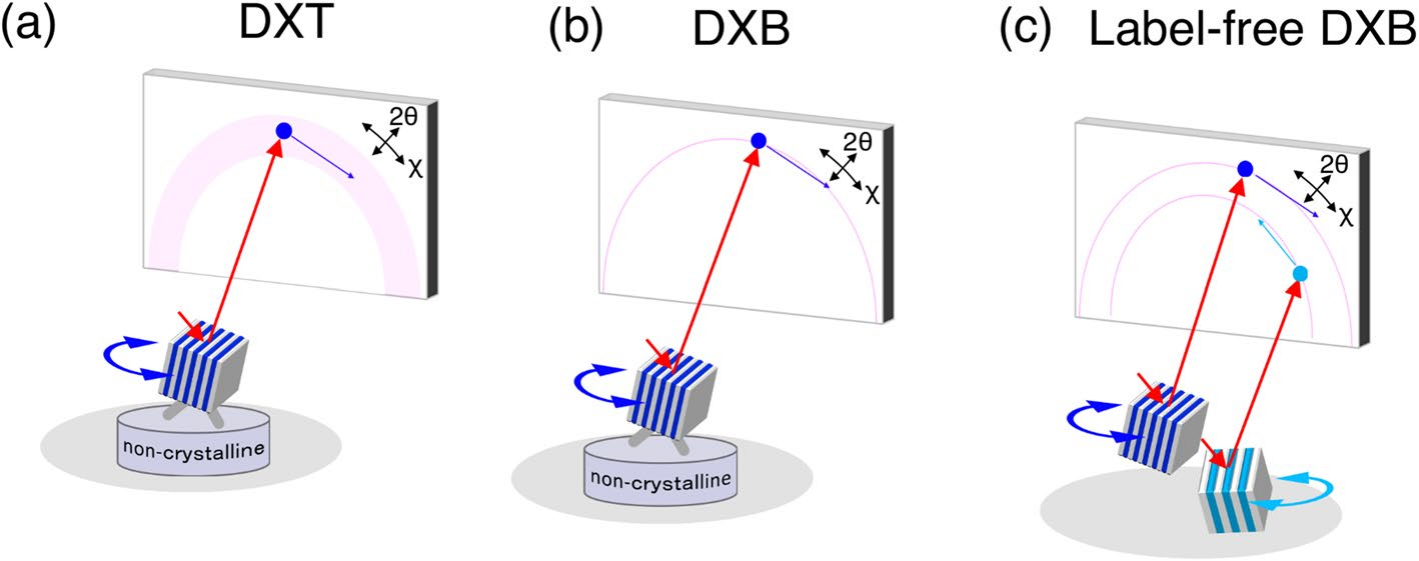
Diffracted X-ray Tracking (DXT) and Diffracted X-ray Blinking (DXB). Schematic illustration of (**a**) DXT, (**b**) DXB and (**c**) label-free DXB for crystalline substances.

X-ray diffraction measurements significantly contribute to determining stable phases before and after physical and chemical reactions in multiple grains. Time-resolved X-ray observations are also used to identify quantitative changes in stable phases, but such observations do not monitor dynamic motion^1–5^. The first observation of the motions of nanocrystals at the microsecond resolution was performed using a broadband X-rays for the investigation of single protein molecules. This technique, termed “Diffracted X-ray Tracking” (DXT), can detect tilting and rotational motions of protein-labelled gold nanocrystals as the angular velocity with milli-radian accuracy. The trajectory of a Laue diffraction spot from a nanocrystal reflects those motions of protein molecules (Fig. 1a). Tilting and rotational motions are assigned from the θ and χ direction movements of the diffraction spots. DXT can measure those motions related to the intramolecular function of membrane proteins below Å^6–11^, but requires a broad energy bandwidth of incident X-rays. Recently, a DXT-like measurement was shown to be possible with the use of a monochromatic X-ray beam. X-ray diffraction spots from moving nanocrystals are observed to cycle in and out of the Bragg condition (Fig. 1b). The movements of diffraction spots appear to behave in an on/off (blinking) fashion at the Debye–Scherrer ring, which we term “Diffracted X-ray Blinking” (DXB)^12,13^. The tilting and rotational motions of a protein molecule labelled with nanocrystals can be evaluated as its exponential relaxation by ACF. The relaxation time is defined as the ACF decay constant, which is closely related to the fluctuation of the diffraction intensity with respect to grain motions. DXT and DXB provide benefits for observing the tilting and rotational motions of biomolecules at the atomic scale by using X-rays and nanocrystal probes, but they require the attachment of the nanocrystal probe to the biomolecule of interest.

Here, we apply DXB to directly measure the tilting and rotational motions of single-crystal grains in silver halides during photoreactions. In situ DXB is referred to as “Label-free DXB”. The diffraction of multiple nanocrystals with different structures can also be simultaneously observed over time (Fig. 1c). We show the grain motions of silver halides during photolytic reactions induced by X-ray photons. Furthermore, we also show the grain motions and lattice spacing changes of the produced Ag. The ACF decay constant of the produced Ag correlates with that of silver halides. The relationship of the grain motions between silver halides and Ag produced during photolytic reactions is discussed.

## Results and discussion

**Figure 2:**
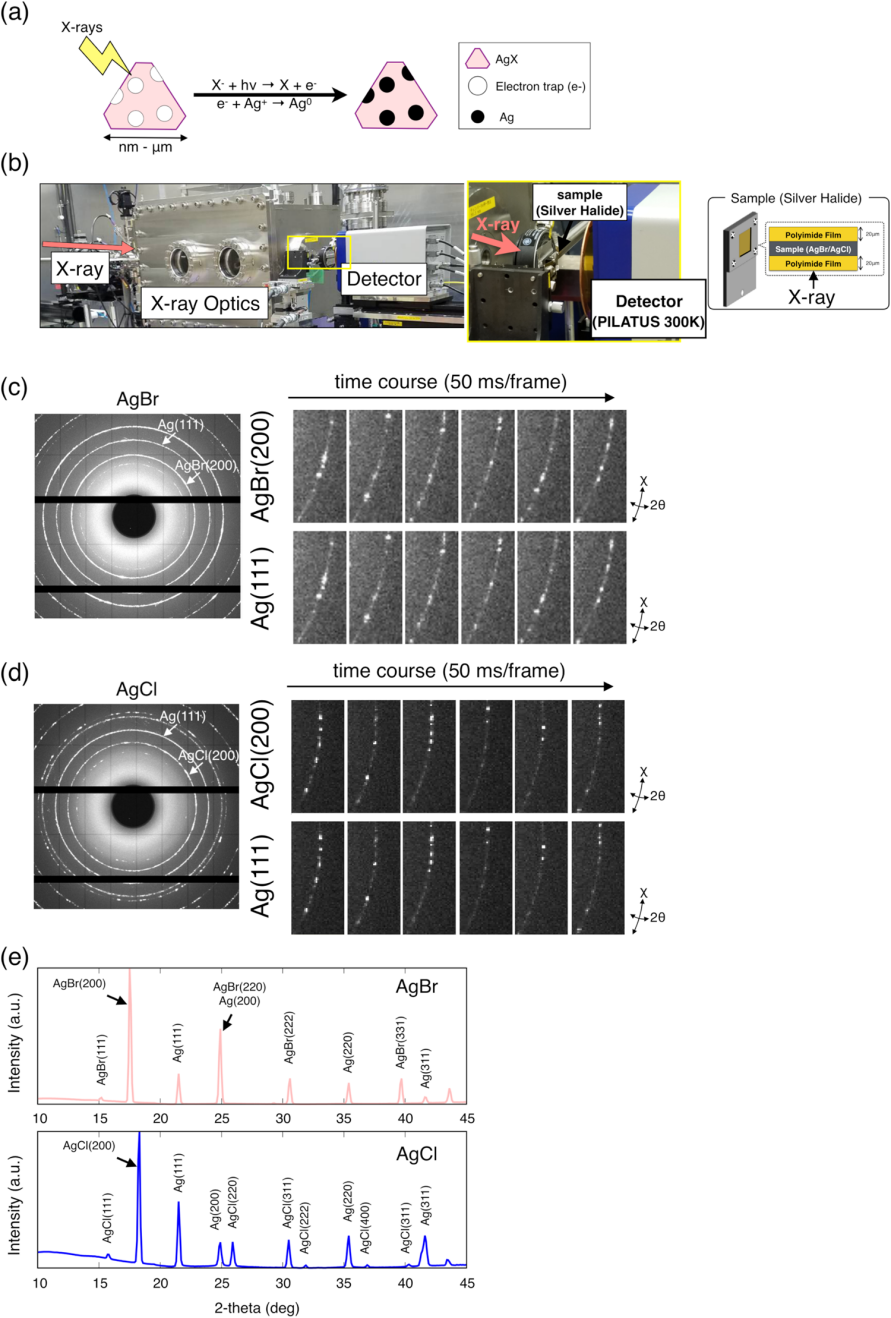
DXB measurement for silver halides. (**a**) Schematic illustration of the photoinduced chemical reaction with silver halide. (**b**) DXB setup at the BL39XU in SPring-8 (Japan). Monochromatic X-rays (E = 15.2 keV) were focused by two K–B mirrors. The beam size was 0.52 × 0.77 μm. The sample-to-detector distance was 85 mm, and 2000 time-resolved diffraction images from silver halide nanocrystals immobilized on the polyimide film were recorded with a 2D photon-counting detector (Pilatus 300 K, Dectris Switzerland). (**c**, **d**) X-ray diffraction images of silver halides from the DXB measurement with monochromatic X-rays. The exposure time per frame and interval time were set to 50.0 and 53.0 ms, respectively. (**e**) Two-theta diagrams in AgBr, AgCl and Ag. Diffraction peaks are assigned to each plane of the face-centred cubic (fcc) lattice structure in AgBr, AgCl, and Ag.

Silver halides, such as AgBr and AgCl, are often used in photographic films. The material properties of AgBr and AgCl crystal grains change through photoinduced chemical reactions. Photoreaction of silver halide produces negatively charged electrons and positively charged silver ions. These ionic forms float freely in the crystalline matrix. Sensitivity specks attract silver ions through trapped electrons, and metallic silver grains are produced around the sensitivity specks (Fig. 2a)^14–18^. Silver halides can be directly monitored by diffraction because of their crystal grain structure. Time-resolved XRD images were obtained using a high-speed PILATUS detector^19^ at the BL39XU beamline in SPring-8 (Fig. 2b). The entire dynamic process of photoreaction involving silver halides during X-ray irradiation was characterized by measuring the extremely subtle changes in the X-ray diffraction intensity with a 50 ms time resolution. We observed the non-annealed AgBr, annealed AgBr, non-annealed AgCl and annealed AgCl during photoinduced reaction. The median grain diameters of non-annealed AgBr, annealed AgBr, non-annealed AgCl and annealed AgCl were 104, 216, 211 and 890 nm, respectively (Fig. S1a and Table S1). The diffraction spots in Debye–Scherrer rings from AgX (Br or Cl) were directly observed during X-ray exposure. Diffraction rings of metallic silver were also observed after a few frames (Fig. 2c–e). Considering the X-ray beam size (0.52 × 0.77 μm), several large diffraction-spots with high intensity were probably diffracted from single AgX grains.

**Figure 3:**
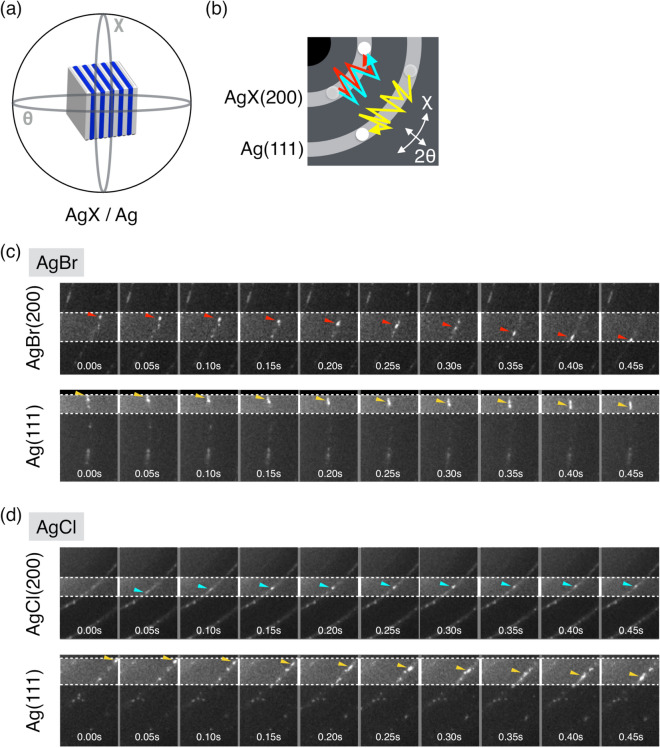
Movements of the diffraction spots in silver halide AgX(200) and silver Ag(111). (**a**) Schematic illustration of the detection of internal motions of silver halides and silver by DXB. (**b**) Schematic illustration of the diffraction spot movement in AgX(200) and Ag(111). (**c**, **d**) Movement of the diffraction spots on AgX(200) and Ag(111) in AgBr and AgCl, respectively.

Interestingly, the diffraction intensity of Ag(111) gradually increased while that of AgBr(200) or AgCl(200) decreased with time (Fig. S2). Long-term intensity changes probably reflect the photolytic chemical reaction. Additionally, individual diffraction spots of AgX and elemental Ag seemed to move in the θ and χ directions (Movies S1 and S2). Based on our experience with DXT^6–11^, the movements of diffraction spots represent the tilting and rotational motions of the nanocrystals (Fig. 3a,b). Actually, the diffraction spots of AgX(200) and Ag(111) were observed to move sequentially in the χ direction with swinging in the θ direction (Fig. 3c,d). These behaviours of the diffraction spots indicate that AgX and Ag rotate with tilting.

**Figure 4:**
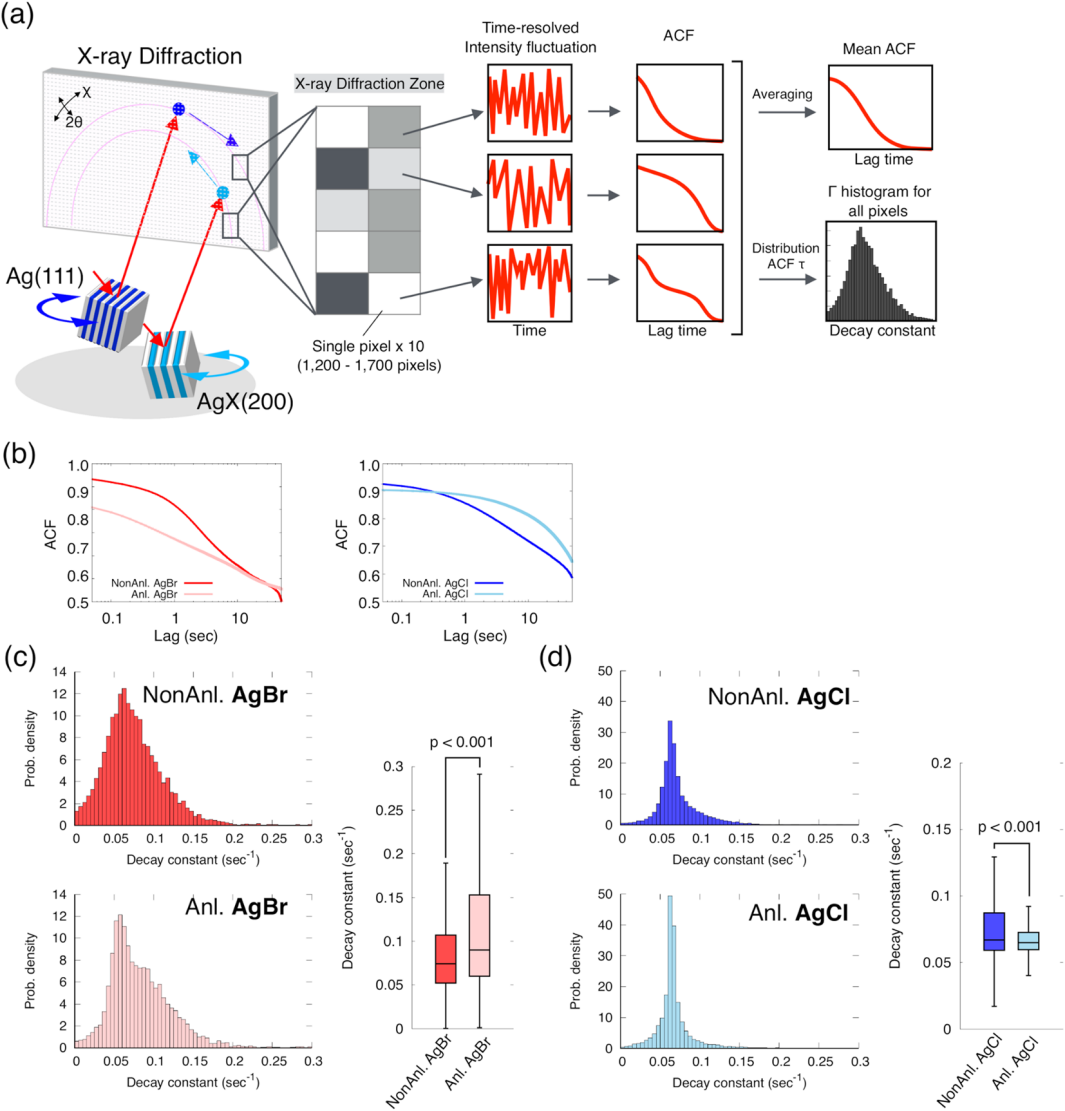
ACF analyses in silver halides AgX(200). (**a**) Concept of in situ DXB for dynamic measurement of crystal grains. The time-resolved intensity fluctuation in the X-ray diffraction zone is analysed by a single-pixel ACF. (**b**) Mean ACF curves for AgBr and AgCl. (**c**, **d**) Histogram and boxplot of decay constants. The distribution of the ACF decay constants was compared between non-annealed and annealed samples of AgBr and AgCl. The boxes show the median and first and third quartiles. The nonparametric Brunner–Munzel test was performed to compare two samples.

Our DXB analysis, without the tracking of diffraction spots, analyses the time-resolved fluctuations of the diffraction intensity using the ACF. Our previous study showed that the ACF decay constant of protein molecules with nanocrystals correlates with the angular velocity^12^. The time trajectory of the diffraction intensity of silver halides reflects the grain motions during the photolysis chemical reaction. The tilting and rotational motions are represented as fluctuations of the diffraction intensity (Fig. 3c,d). The photolysis chemical reaction of silver halides is represented as long-term intensity decay (Fig. S2). To evaluate the motions of the non-annealed and annealed crystal grains in AgBr and AgCl, we calculated the ACF decay constant from the intensity fluctuation of AgX(200) at the level of single pixel (sp) ACF after correcting for long-term decay (Figs. 4a and S3). The decay constants of the mean ACF for non-annealed AgBr, annealed AgBr, non-annealed AgCl and annealed AgCl were 0.105, 0.152, 0.071 and 0.041 s^−1^, respectively (Fig. 4b and Table S2). These results indicate that the motions of crystal grains of non-annealed AgBr are faster than those of annealed AgBr, whereas those of non-annealed AgCl are slower than those of annealed AgCl. The mean ACF supports an intuitive understanding of the differences in the motions of crystal grains, but the examination of individual traces from each pixel in the AgX reveal various motions not visualized in the mean ACF curve. We determined the distribution of the ACF decay constants by sp-ACF analysis of AgX (200) (Fig. S3). The median ACF decay constants of non-annealed AgB, annealed AgBr, non-annealed AgCl and annealed AgCl were 0.0742, 0.0899, 0.0669, and 0.0649 s^−1^, respectively (Table S3). The relationships among the samples approximately correspond to the mean ACFs. Furthermore, the ACF decay constant of annealed AgBr showed a broader distribution histogram than those of non-annealed AgBr (Fig. 4c). By contrast, the ACF decay constant of annealed AgCl displayed a narrower histogram than that of non-annealed AgCl (Fig. 4d). A broad distribution suggests variability in the tilting and rotational motions of individual AgX crystals, and a narrow distribution reflects uniform tilting and rotational motions of individual AgX crystals. Single-pixel ACF analysis revealed significant differences in the distributions of the ACF decay constants between annealed and non-annealed AgX (200).

**Figure 5:**
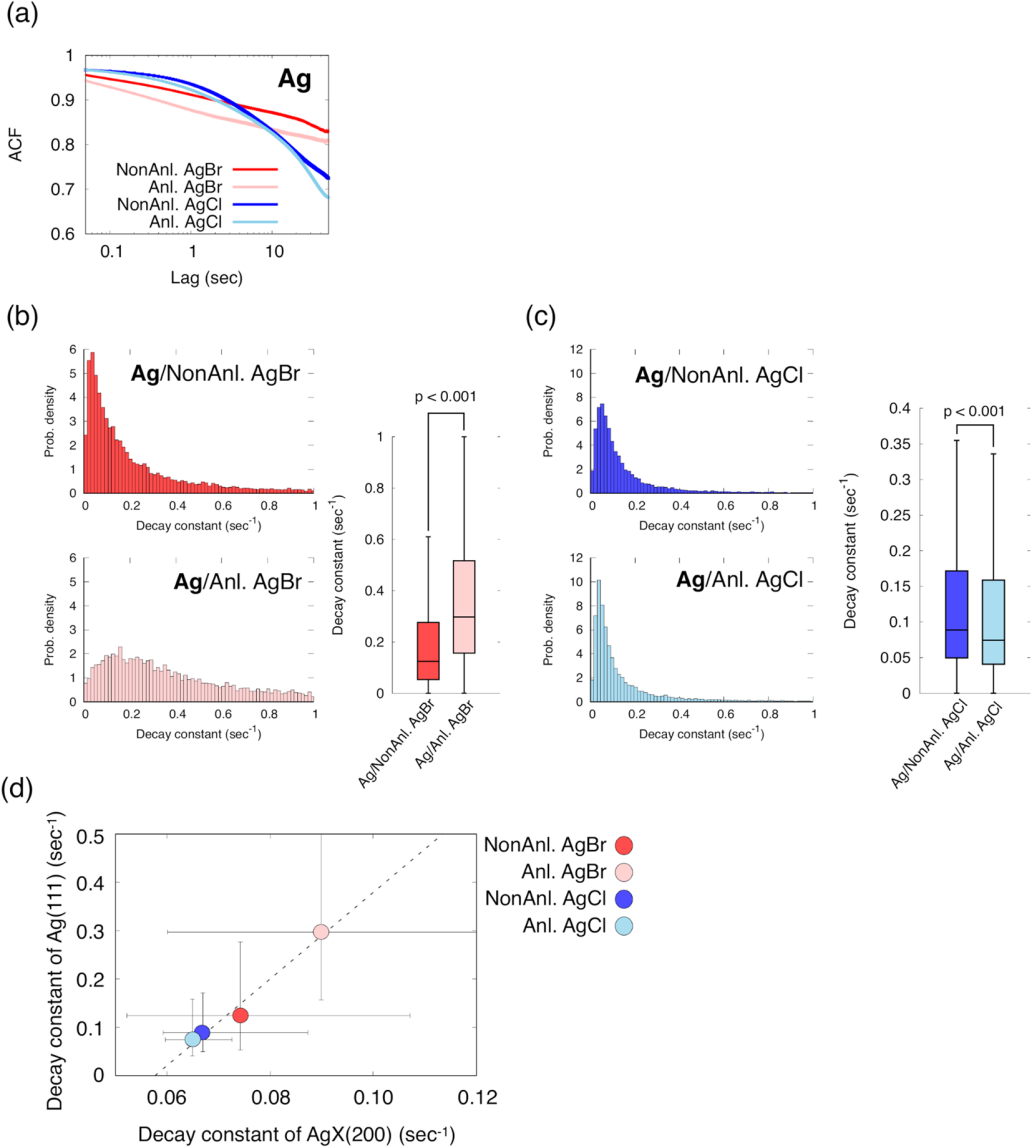
ACF analyses in metallic silver Ag(111). (**a**) Mean ACF curves for Ag for each AgX sample. (**b**, **c**) Histogram and boxplot of the decay constant of Ag. The distribution of the ACF decay constants was compared between non-annealed and annealed samples of Ag/AgBr and Ag/AgCl. The boxes show the median and first and third quartiles. The nonparametric Brunner–Munzel test was performed to compare two samples. (**d**) A correlation of the ACF decay constants between AgX(200) and Ag(111). The dashed line was determined by linear regression.

Metallic silver was produced from AgX by photolysis (Fig. 2a). The intensity of Ag(111) gradually increased during X-ray exposure (Fig. S2 and Movies S1 and S2). After correcting for long-term intensity changes, we analysed the blinking activity of Ag(111) using ACF. The mean ACF decay constants of Ag(111) with AgBr, annealed AgBr, AgCl and annealed AgCl were 0.071, 0.159, 0.070, and 0.060 s^−1^, respectively (Fig. 5a and Table S4). The median values of the ACF decay constants of non-annealed AgBr, annealed AgBr, non-annealed AgCl and annealed AgCl were 0.1245, 0.2972, 0.0889 and 0.0745 s^−1^, respectively (Table S5). The distribution of the ACF decay constant of Ag(111) was significantly different between the non-annealed and annealed samples (Fig. 5b,c). Focusing on the behaviours of the diffraction spots, the spots in Ag(111) moved in the θ and χ directions, indicating that those movements represented tilting and rotational motions (Fig. 3c,d). In addition, the diffraction spots seemed to be sometimes moved between Ag(111) and Ag(200) (Fig. S4). Miao and colleagues reported that the movement of Ag is the lattice spacing change of Ag^20^. Lattice spacing changes with grain motions occurred between Ag(111) and Ag(200). The phenomenon was observed in Ag but not in AgX(200), suggesting that Ag is newly formed with the unstable lattice structure during crystal formation process. The ACF decay constant of silver probably contains changes in the diffraction intensity of grain motions accompanied by lattice spacing changes.

Interestingly, the ACF decay constant of Ag(111) correlates with the constant of AgX(200) diffraction (Figs. 5d and S5). The diffraction intensity of AgX(200) gradually decreased and reached saturated equilibrium after sustained X-ray exposure. On the other hand, the diffraction intensity of Ag(111) increased and reached saturated equilibrium during X-ray exposure (Fig. S2). The diffraction spots on Ag(111) showed grain motions and lattice spacing changes. The lattice spacing changes were a relatively rare event, and simultaneously accompanied the tilting and rotational motions (Fig. S4). The tilting and rotational motions might be the dominant factor for the change in diffraction intensity of Ag. Thus, the relationship of the ACF decay constant between AgX(200) and Ag(111) suggests that Ag grain motions reflect an AgX–Ag complex.

The time trajectory of the diffraction intensity in silver halides shows grain motions during the photolysis chemical reaction. DXB for silver halides and silvers could monitor the tilting and rotational motions, and the lattice spacing change that occurred during the photolysis chemical reaction. Single-pixel ACF analysis of AgX(200) demonstrates the motions of single grains AgBr and AgCl. In addition, the ACF decay constant of the produced Ag single grains correlated with that of AgX.

We evaluated whether ACF decay constant correlates with grain size. The ACF decay constants of AgX did not correlate with the diameter of the multiple grains (Fig. S1b). Furthermore, we analysed the full-width at half medium (FWHM) of AgX (200) diffraction from the 2-theta diagram. Generally, the FWHM of a diffraction peak based on the Scherrer equation is often used to determine crystallite sizes from XRD spectra^21^ (Fig. S6a). The FWHMs of AgX(200) on AgBr, annealed AgBr, AgCl and annealed AgCl were 0.2349°, 0.2567°, 0.2327° and 0.2307°, respectively (Fig. S6b and Table S6). The relationship between the FWHM of AgX (200) diffraction and the ACF decay constants of each grain did not show a clear correlation (Fig. S6c). The FWHMs of Ag(111) on AgBr, annealed AgBr, AgCl and annealed AgCl were 0.2315°, 0.2675°, 0.2855° and 0.2920°, respectively (Fig. S6d and Table S7). The FWHM of non-annealed AgBr (or AgCl) is larger than that of the annealed forms. The FWHM of Ag did not correlate with the ACF decay constant (Fig. S6e). We did not find the clear correlation between grain motion and the grain size.

Here, we attempt to estimate the rotational diffusion coefficient of silver halides during photoreaction. The limitation of the Pilatus detector exposure-time performance, 50 ms, is the fastest. Although the limited time resolution cannot exactly monitor the photolysis reaction, it is useful to quantitatively determine the detection accuracy of DXB in real-time and real-space. The ACF decay constant represents the rotational diffusion time for tilting motion that the diffraction spot takes to move a single pixel. The angular widths of AgBr(200) and AgCl(200) for a single pixel were 0.03077 and 0.02840 Å, respectively. By multiplying the angular width squares times the ACF decay constant, the rotational diffusion coefficient was estimated using Eq. (4) (Fig. S7). Because the diffraction intensity of Ag(111) contains grain motions and lattice spacing changes (Figs. 3c,d and S4), we did not estimate the rotational diffusion coefficient of metallic silver. The rotational diffusion coefficients of the silver halides were estimated at approximately 0.1 to 0.3 pm^2^/s in the θ direction (Table S8), indicating that DXB spatially detects atomic-scale motion.

The structure and dynamics of grains are valuable for understanding material properties. The time-resolved information obtained from in situ DXB includes significant dynamic information, such as the size and motion speed of both reaction residues and generated microcrystals and the individual independence and shape changes of the interface around crystals. In the future, DXB with a high-speed camera will be able to detect nanocrystal structures on the order of microseconds. Our novel technique leads to a deeper understanding of the mechanisms underlying crystal dynamic motion and function.

## Methods

### Sample preparation for silver halides

AgBr and AgCl nanograins were fabricated via epitaxial growth on a cleaved polyimide film (area: 7 × 7 mm^2^) in 10^−4^ Pa vacuum. The shape and quality of the silver halide nanocrystals were confirmed using atomic force microscopy on 1,000 particles inside a 100 μm^2^ polyimide film. Annealed samples were heated to 300° in a high-temperature incubator.

### Diffracted X-ray blinking (DXB)

DXB measurements were performed using the BL39XU beamline (SPring-8, Japan). The X-ray beam was monochromatized to 15.2 keV using a Si 111 double-crystal monochromator. Monochromatic X-rays were focused to a spot of ∼ 0.52 μm × 0.77 μm using K–B mirrors. The sample-to-detector distance was 85 mm, and 2,000 time-resolved diffraction images from silver halide nanocrystals immobilized on the polyimide film were recorded on a 2D photon-counting detector (Pilatus 300 K, Dectris Switzerland). The exposure time per frame and interval time were set to 50.0 and 53.0 ms, respectively.

### ACF analysis and evaluation of the ACF decay constant

The intensity values of AgBr(200), AgCl(200) and Ag(111) in each pixel, without the intermodular rectangular area of the detector, were extracted by ImageJ software. The time-resolved intensity fluctuations of the AgBr(200), AgCl(200) and Ag(111) trends over the long term were determined during the photolysis reaction (Fig. S2). Such trends were removed by the fitted nonlinear curve with a third-order function (Fig. S3). The time-resolved intensity fluctuation of each pixel was computed to evaluate rotational motions using the following autocorrelation function (ACF)^12^:

1 $$\begin{array}{*{20}c} {I\left( {\varvec{\tau}} \right) = \frac{{\left\langle {{\mathbf{I}}\left( {\varvec{\tau}} \right){\mathbf{I}}\left( {{\mathbf{t}} + {\varvec{\tau}}} \right)} \right\rangle }}{{ \left\langle {{\mathbf{I}}\left( {\mathbf{t}} \right)^{2} } \right\rangle }}} \\ \end{array}$$

where I(t) represents the diffracted photon intensity. The brackets 〈〉 indicate time-averaged values. The computed ACF was fitted to single exponential curves by ACF(t) = Aexp(− Гt) + y, where A is the amplitude, y is the conversional value and Г is the decay constant. Parameters A and y were determined from computed ACF data. Parameter Г was optimized to fit the ACF curve by nonlinear least squares with the Levenberg–Marquardt algorithm in gnuplot software. Several ACF curves did not show a single exponential decay because the signal-to-noise ratio was low. Thus, we chose decay constants to satisfy the following conditions: (I) 0 < y, 0 < A and 0 < Г^12^ and (II) residual values between the fitted and actual ACF curves of less than 0.5. These calculations were performed for ACF data for all pixels. The mean ACF curves and distributions of the decay constants were generated from selected 6,000–15,000 pixels through the above ACF filter process (see Tables S3 and S5 for details).

The distributions of the ACF decay constants were statistically analysed with the non-parametric Brunner–Munzel test^22^. This test evaluated the equality of the means between non-annealed and annealed samples and was used because the distributions were not Gaussian distributions based on the Shapiro–Wilk test.

### Crystal grain size by atomic force microscopy (AFM)

The sizes of the crystal grains in non-annealed AgBr, annealed AgBr, non-annealed AgCl and annealed AgCl were measured by AFM on approximately 100 particles between 2 and 10 μm^2^ polyimide films. The grain sizes from the AFM image data were analysed by Gwyddion ver. 2.53 (http://gwyddion.net).

### Full-width at half maximum (FWHM) evaluation in Debye–Scherrer ring

For AgBr(200), AgCl(200) and Ag(111), we estimated the crystalline size from the FWHM of the Debye–Scherrer ring. First, high-signal images from summing 2,000 frames were used to draw 2θ diagrams using FIT2D software. The diffraction peaks of AgX(200) and Ag(111) were fitted with standard Gaussian curves and evaluated using the FWHM of the Gaussian parameters.

### The rotational diffusion coefficient

DXB measurement only detects the tilting and rotational motions. The diffraction spots were not moved by the translational movement of crystal grains. Because the diffraction intensity does not change, the translational motion is neglected in ACF analysis for DXB (Fig. S7). The rotational diffusion coefficient *D*_*R*_ of a single colloidal sphere with radius *r* is defined by the Stokes–Einstein–Debye (SED) relation^23–25^ as follows:

2 $$\begin{array}{*{20}c} {D_{R} = \frac{{{\text{kT}}}}{{8\pi \eta r^{3} }}} \\ \end{array}$$

where k is the Boltzmann constant, T is the temperature, and η is the viscosity. A mean square displacement <φ_θ_^2^> for tilting rotational diffusion is defined as:^26,27^

3 $$\begin{array}{*{20}c} {\varphi_{\theta }^{2} = \frac{{4{\text{kT}}}}{{8\pi \eta r^{3} }}t = 4D_{R} t} \\ \end{array}$$

The ACF decay constant Г is directly related to the rotational diffusion coefficient of single grains *D*_*R*_ as expressed in the following equation.

4 $$\begin{array}{*{20}c} {D_{R} = \frac{{\varphi_{\theta }^{2} \Gamma }}{4}} \\ \end{array}$$

The rotational displacement φ_θ_ was calculated by the angular width of the 2θ diagram. The angular widths of AgBr(200) and AgCl(200) for 1 pixel in the XRD images were approximately 0.03077 and 0.0284 Å, respectively. As shown Eq. (4), rotational diffusion was estimated by multiplying the angular width squares times the ACF decay constant. For this calculation, we used the quartile values of the ACF decay constants.

## Supplementary Information

Supplementary Movie 1.

Supplementary Movie 2.

Supplementary Information.

## Data Availability

The datasets generated during and/or analysed during the current study are available from the corresponding author on reasonable request.
