# Supplementary material for: Tilting and rotational motions of silver halide crystal with diffracted X-ray blinking
Source: Sci Rep. 2021 Mar 5;11:4097. doi: 10.1038/s41598-021-83320-y (PMC7935957; doi:10.1038/s41598-021-83320-y)
Supplement: Supplementary file 3 — Supplementary Information. [file 41598_2021_83320_MOESM3_ESM.pdf]

Supplementary Information for

Tilting and Rotational Motions of Silver Halide Crystal with Diffracted X-ray Blinking

Masahiro Kuramochi, Hiroki Omata, Masaki Ishihara, Sander Øglænd Hanslin, Masaichiro Mizumaki, Naomi Kawamura, Hitoshi Osawa, Motohiro Suzuki, Kazuhiro Mio, Hiroshi Sekiguchi, and Yuji C. Sasaki.

Masahiro Kuramochi
Email: masahiro-kuramochi@edu.k.u-tokyo.ac.jp

Yuji C. Sasaki
Email: ycsasaki@edu.k.u-tokyo.ac.jp

This file includes:

Figs. S1 to S7
Tables S1 to S8

Correspondence to: masahiro-kuramochi@edu.k.u-tokyo.ac.jp, ycsasaki@edu.k.u-tokyo.ac.jp

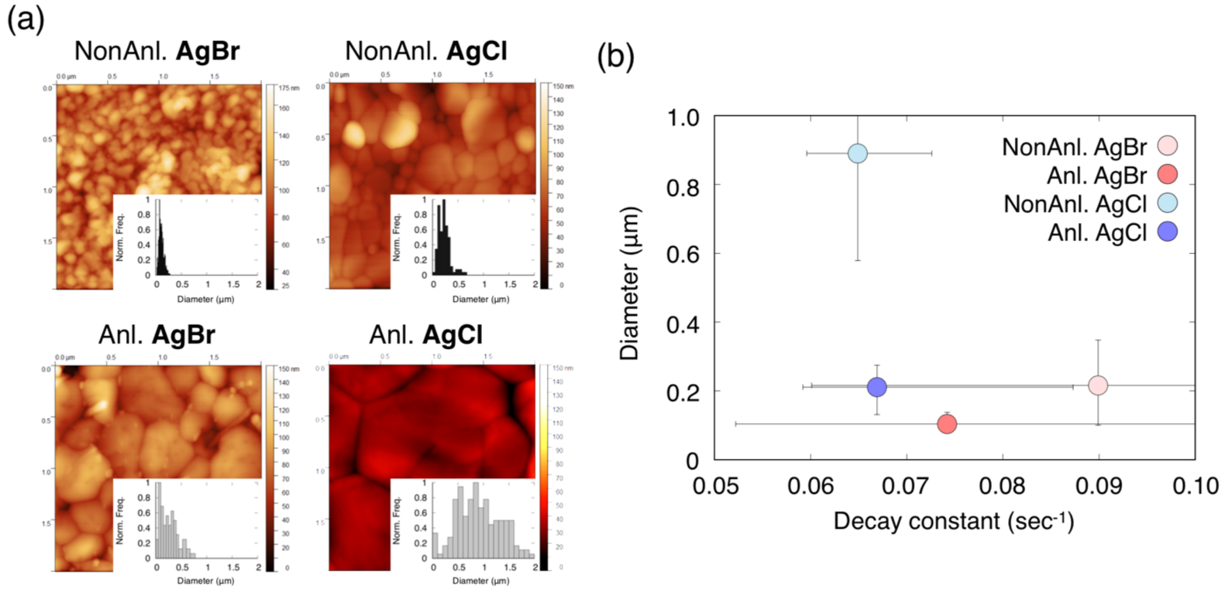

Fig. S1. Grain diameters of silver halides from AFM observation.

(a) Observation of the grain diameters by AFM. The inset panels show the distribution of grain size. (b) Grain diameter vs ACF decay constant.

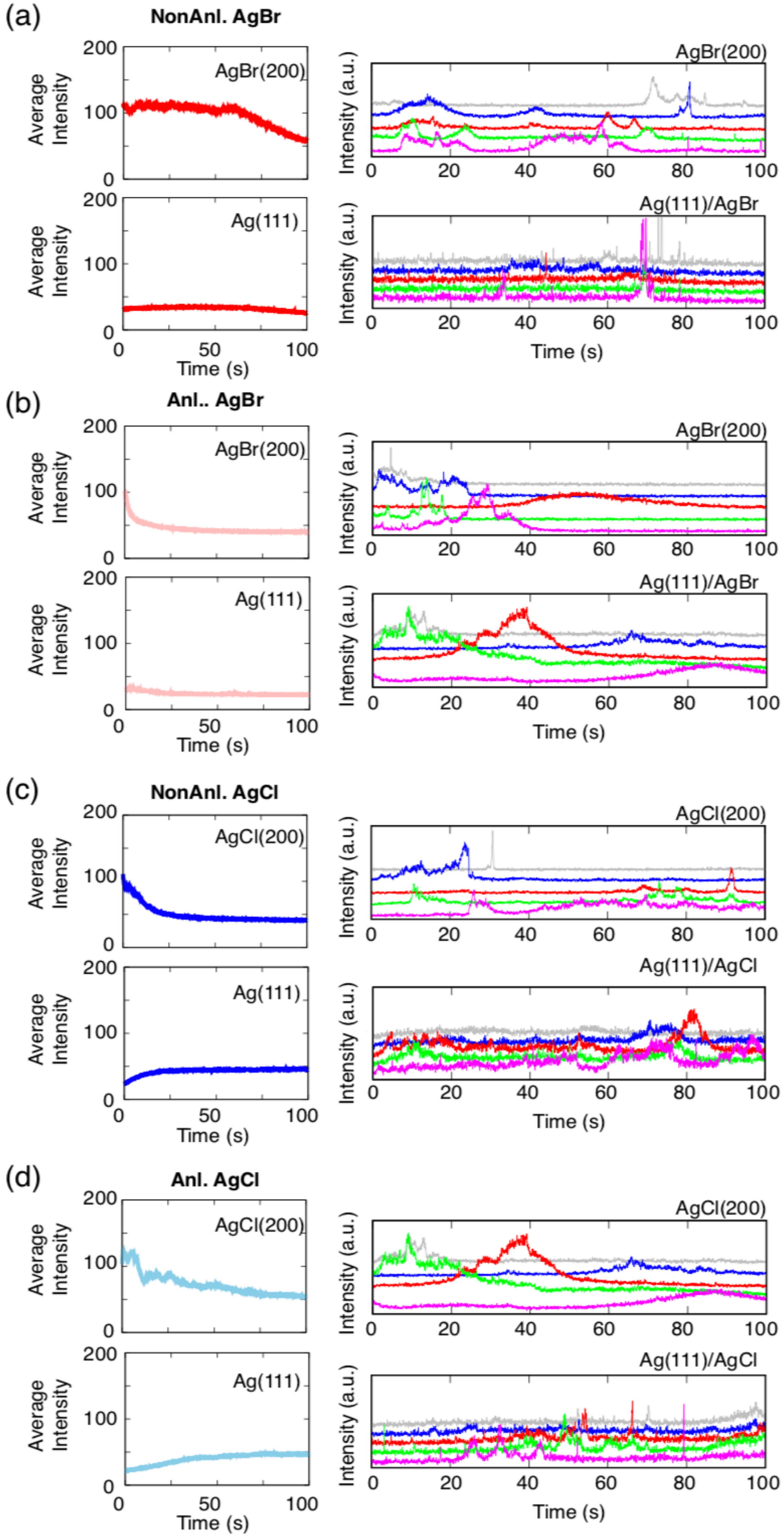
(a)
NonAnl. AgBr
AgBr(200)
Ag(111)
AgBr(200)
Ag(111)/AgBr
Average Intensity
Intensity (a.u.)
Time (s)
(b)
Anl.. AgBr
AgBr(200)
Ag(111)
AgBr(200)
Ag(111)/AgBr
(c)
NonAnl. AgCl
AgCl(200)
Ag(111)
AgCl(200)
Ag(111)/AgCl
(d)
Anl. AgCl
AgCl(200)
Ag(111)
AgCl(200)
Ag(111)/AgCl

Fig. S2. Intensity changes of AgBr(200), AgCl(200) and Ag(111).

(a-d) Averaged intensity changes of AgBr(200), AgCl(200) and Ag(111). The AgBr(200) and AgCl(200) intensities gradually decreased during X-ray exposure. By contrast, the Ag(111) intensity was gradually increased, indicating that Ag is produced from AgX through a photoinduced chemical reaction (left). Intensity changes of individual pixels in AgBr(200), AgCl(200) and Ag(111) after removal of the long-term trend (right). The trends were removed with fitted nonlinear curves with a three-order function.

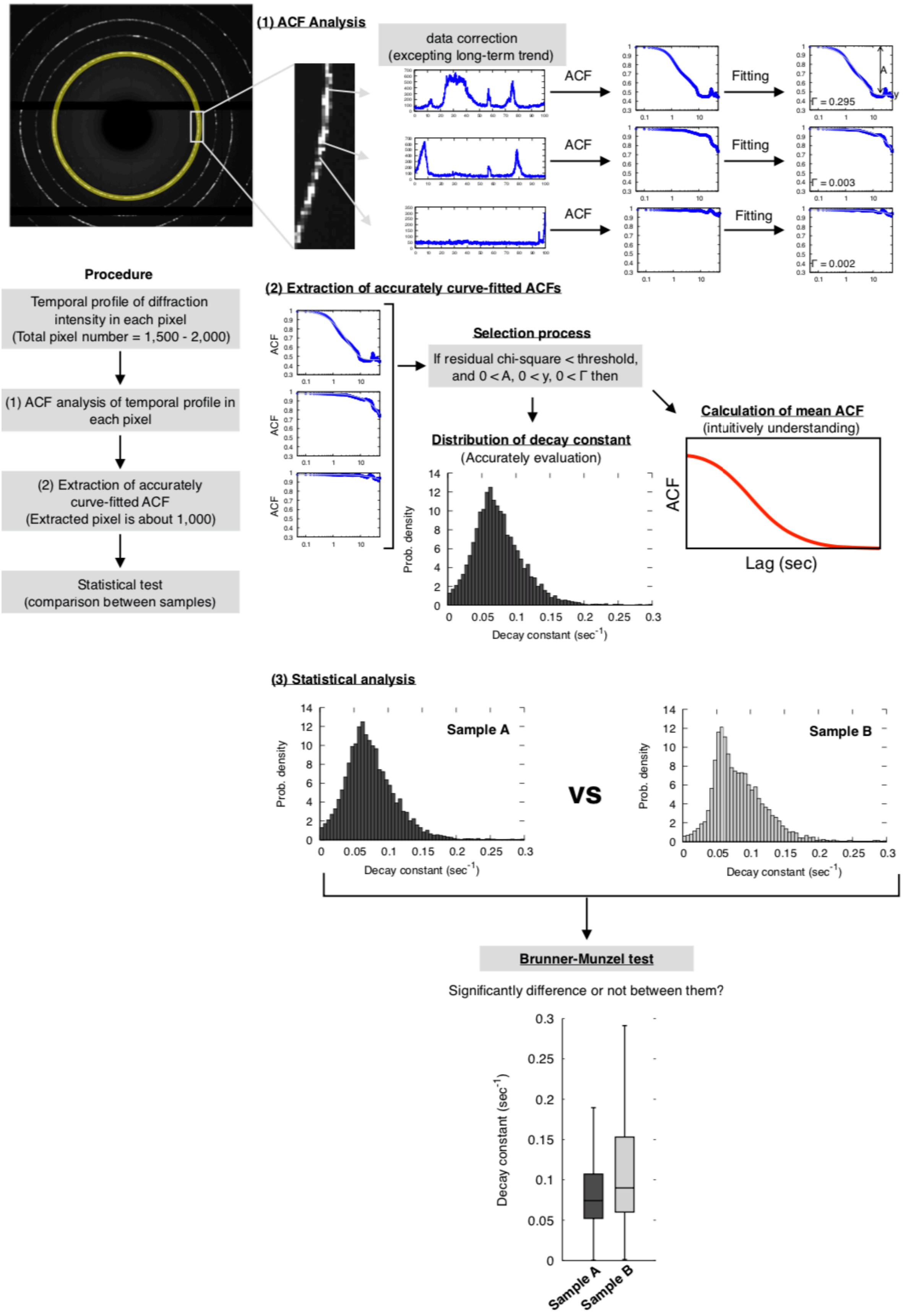
(1) ACF Analysis
data correction
(excepting long-term trend)
ACF
Fitting
A
y
Γ = 0.295
Γ = 0.003
Γ = 0.002
Procedure
Temporal profile of diffraction intensity in each pixel
(Total pixel number = 1,500 - 2,000)
(1) ACF analysis of temporal profile in each pixel
(2) Extraction of accurately curve-fitted ACF
(Extracted pixel is about 1,000)
Statistical test
(comparison between samples)
(2) Extraction of accurately curve-fitted ACFs
Selection process
If residual chi-square < threshold, and 0 < A, 0 < y, 0 < Γ then
Distribution of decay constant
(Accurately evaluation)
Prob. density
Decay constant (sec-1)
Calculation of mean ACF
(intuitively understanding)
ACF
Lag (sec)
(3) Statistical analysis
Sample A
Sample B
VS
Brunner-Munzel test
Significantly difference or not between them?
Decay constant (sec-1)
Sample A
Sample B

Fig. S3. Schematic illustration of the autocorrelation method.

The ROI was first chosen from Debye–Scherrer rings (top left). Time autocorrelation was computed from the intensity fluctuations of each ROI pixel after removing long-term trends. The single-pixel ACF was fitted with a single exponential curve. To exclude inaccurate ACFs, accurate ACF curves were individually selected using the parameters and residuals of the fitted model. Selected single-pixel ACFs were used to evaluate the distribution and mean ACF. A non-parametric Brunner–Munzel test[1] was performed to evaluate whether distributions of ACF decay constants between samples were significantly different.

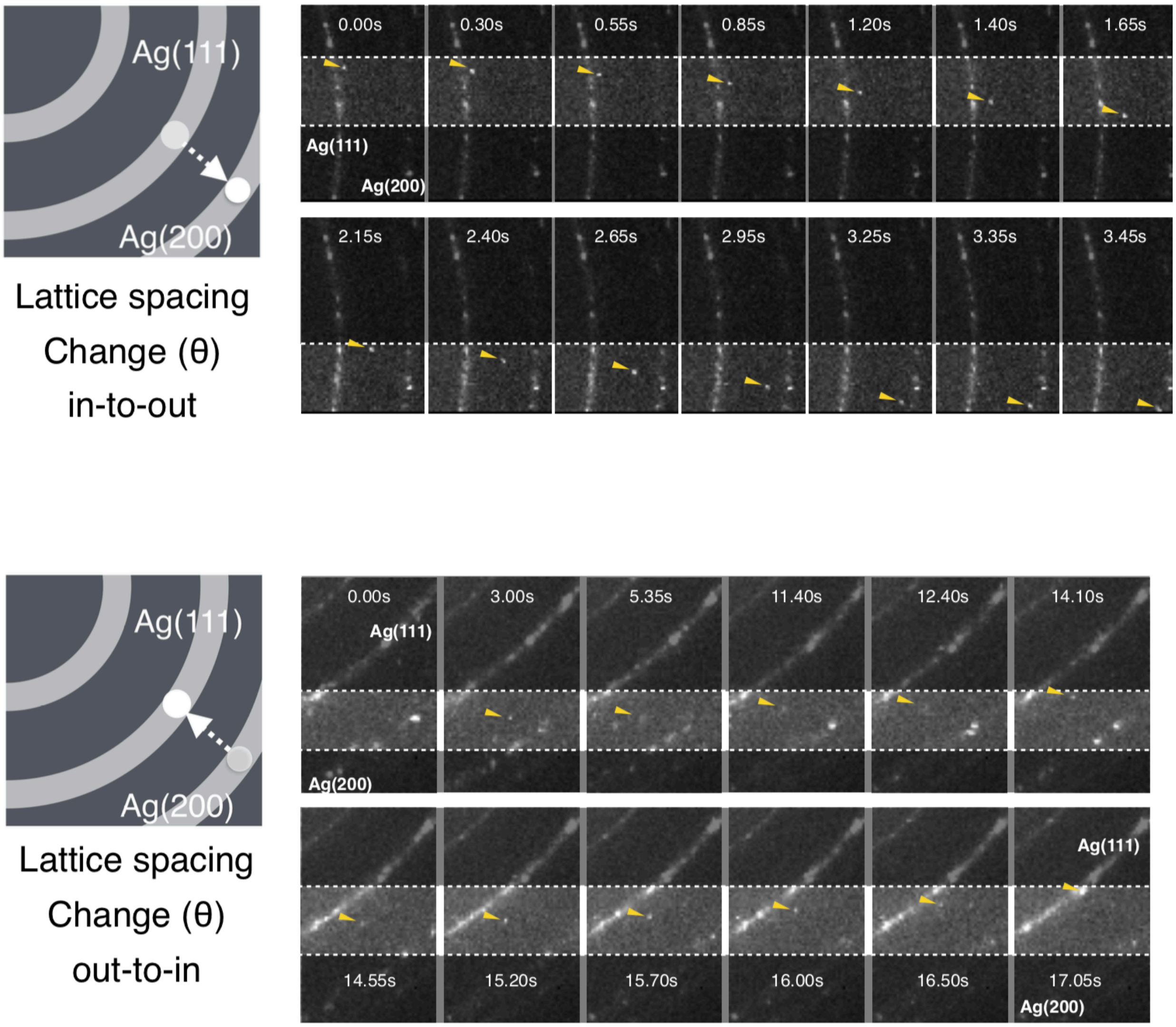

Fig. S4. Lattice spacing changes of Ag(111) in silvers.

(a) Schematic illustration of the diffraction spot movement from Ag(111) to Ag(200) (left). Actual movement of the diffraction spot from Ag(111) to Ag(200) (right). The yellow arrowhead indicates the diffraction spot. (b) Schematic illustration of the diffraction spot movement from Ag(200) to Ag(111) (left). Actual movement of the diffraction spot from Ag(200) to Ag(111) (right). The yellow arrowhead indicates the diffraction spot.

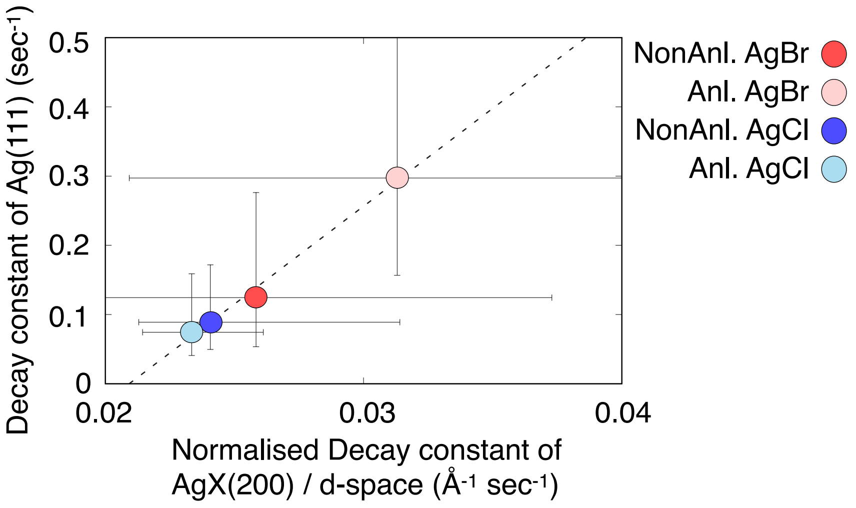

Fig. S5. Correlation of the ACF decay constants between AgX(200) and Ag(111).

The ACF decay constants of AgX(200) were normalised by d-spaces, because there are different between d-spaces in AgBr(200) and AgCl(200). The dashed line was determined by linear regression.

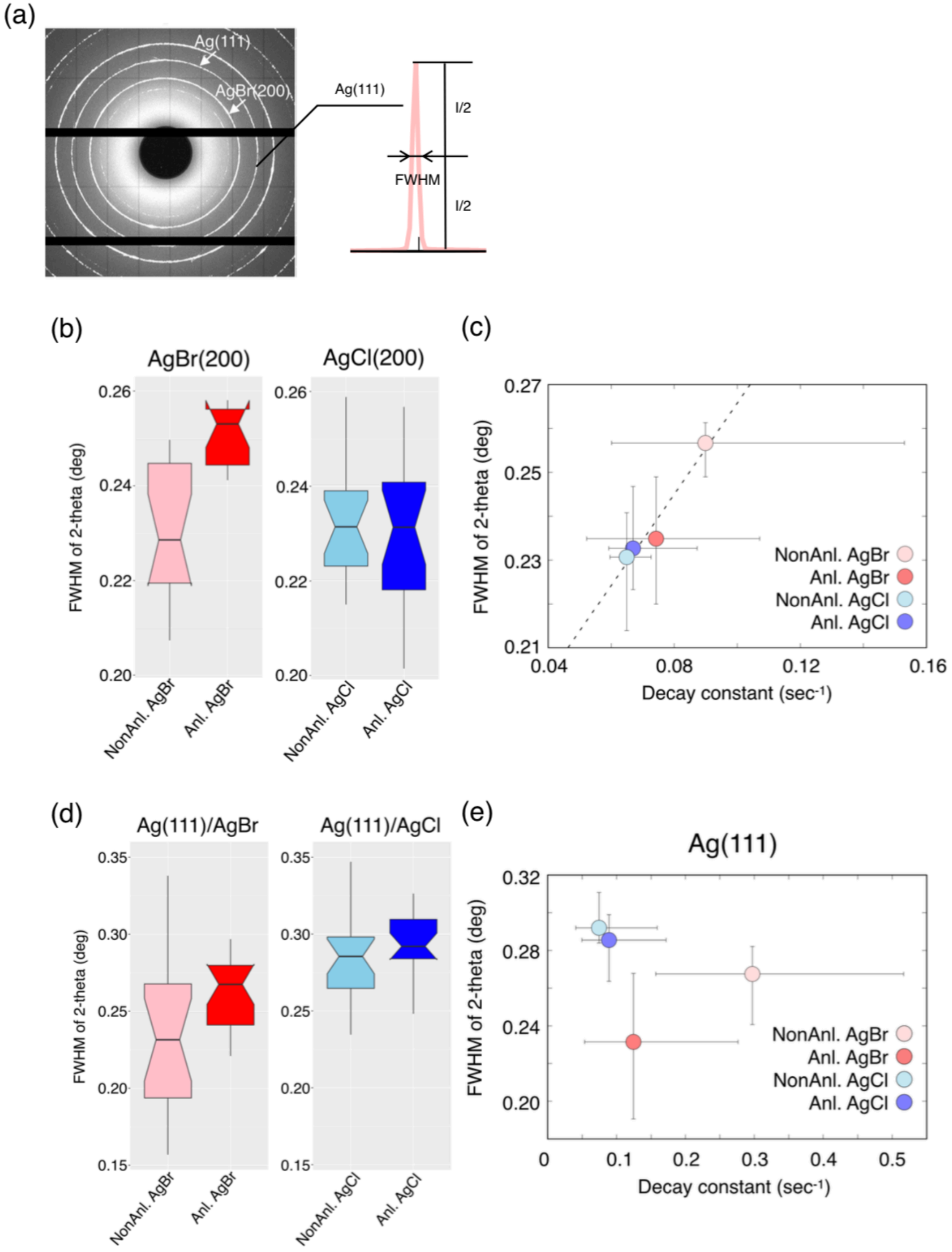
(a)
Ag(111)
AgBr(200)
Ag(111)
I/2
FWHM
I/2
(b)
AgBr(200)
AgCl(200)
FWHM of 2-theta (deg)
0.26
0.24
0.22
0.20
NonAnl. AgBr
Anl. AgBr
NonAnl. AgCl
Anl. AgCl
(c)
0.27
0.25
0.23
0.21
0.04
0.08
0.12
0.16
Decay constant (sec-1)
(d)
Ag(111)/AgBr
Ag(111)/AgCl
0.35
0.30
0.25
0.20
0.15
(e)
Ag(111)
0.32
0.28
0.24
0.20
0
0.1
0.2
0.3
0.4
0.5

Fig. S6. FWHM of 2-theta in AgX(200) and Ag(111).

(a) Schematic illustration of the FWHM analytical method. The FWHM of the 2-theta diagram for AgX(200) and Ag(111) was calculated with fitted Gaussian curves. (b) The distribution of the FWHM of 2-theta in non-annealed and annealed AgX(200) in non-annealed and annealed AgX. The boxes show the median and first and third quartiles. (c) FWHM of 2-theta in AgX(200) vs the ACF decay constant of AgX(200). The dashed line was determined by linear regression. (d) The distribution of the FWHM of 2-theta in non-annealed and annealed Ag(111) in non-annealed and annealed AgX. The boxes show the median and first and third quartiles. (e) FWHM of 2-theta in Ag(111) vs the ACF decay constant of Ag(111). The dashed line was determined by linear regression.

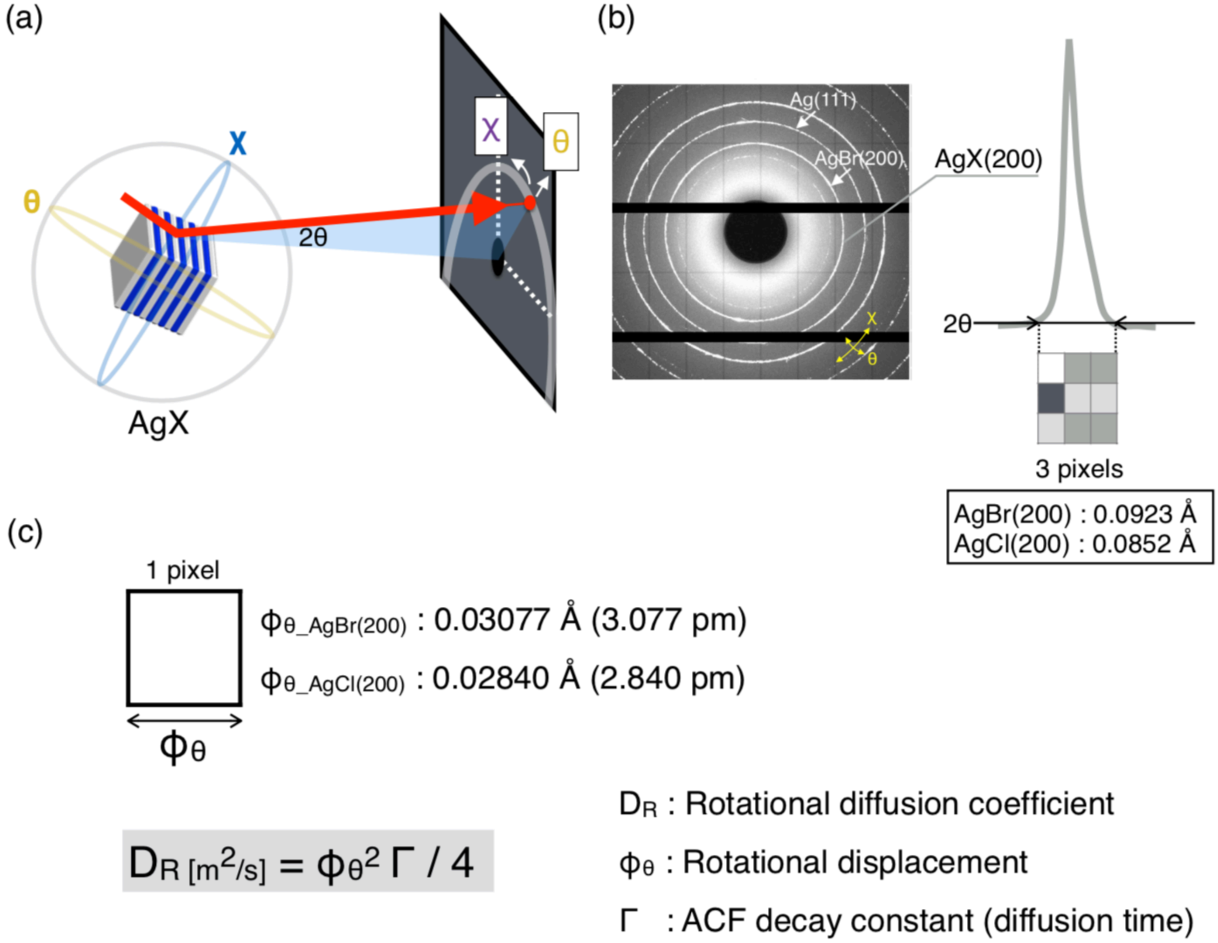

Fig. S7. Rotational diffusion coefficient of silver halides.

(a) Schematic illustration of the diffraction spot movement in DXB. (b) XRD images and 2-theta diagram. (c) Calculation of the rotational diffusion coefficient.

Table S1. Grain diameters from AFM observation.

	25% Quartile (μm)	50% Quartile (μm)	75% Quartile (μm)
NonAnl. AgBr	0.0769	0.104	0.138
Anl. AgBr	0.100	0.216	0.351
NonAnl. AgCl	0.131	0.211	0.277
Anl. AgCl	0.578	0.890	1.2756

Table S2. Mean ACF decay constants and variance of residuals for AgX(200).

	T (s^{-1})	Variance of residuals
AgBr(200)/NonAnl. AgBr	0.105407 ± 0.001389	0.00255846
AgBr(200)/Anl. AgBr	0.152441 ± 0.00213	0.00104844
AgCl(200)/NonAnl. AgCl	0.0711291 ± 0.0006539	0.000743133
AgCl(200)/Anl. AgCl	0.0411328 ± 0.0001668	0.00011905

Table S3. Quartile values of the ACF decay constant histogram for AgX(200).

	25% Quartile (s^{-1})	50% Quartile (s^{-1})	75% Quartile (s^{-1})	Selected pixels/all pixels
AgBr(200)/NonAnl. AgBr	0.0522	0.0742	0.1071	8,318/23,534
AgBr(200)/Anl. AgBr	0.0601	0.0899	0.1530	6,766/23,534
AgCl(200)/NonAnl. AgCl	0.0592	0.0669	0.0873	9,352/23,534
AgCl(200)/Anl. AgCl	0.0596	0.0649	0.0726	11,475/23,534

Table S4. Mean ACF decay constants and variance of residuals for Ag(111).

	T (s^{-1})	Variance of residuals
Ag(111)/NonAnl. AgBr	0.0716014 ± 0.0008804	0.000184688
Ag(111)/Anl. AgBr	0.159606 ± 0.00309	0.000244819
Ag(111)/NonAnl. AgCl	0.0708826 ± 0.0002688	5.92183e-05
Ag(111)/Anl. AgCl	0.060673 ± 0.0002976	0.000152423

Table S5. Quartile values of the ACF decay constant histogram for Ag(111).

	25% Quartile (s^{-1})	50% Quartile (s^{-1})	75% Quartile (s^{-1})	Selected pixels/all pixels
Ag(111)/NonAnl. AgBr	0.0535	0.1245	0.2763	9,869/23,534
Ag(111)/Anl. AgBr	0.1567	0.2972	0.5166	5,906/23,534
Ag(111)/NonAnl. AgCl	0.0496	0.0889	0.1717	14,109/23,534
Ag(111)/Anl. AgCl	0.0408	0.0745	0.1588	15,076/23,534

Table S6. FWHM of 2-theta for AgX(200) diffraction.

	25% Quartile (°)	50% Quartile (°)	75% Quartile (°)
AgBr(200)/NonAnl. AgBr	0.2200	0.2349	0.2490
AgBr(200)/Anl. AgBr	0.2490	0.2567	0.2613
AgCl(200)/NonAnl. AgCl	0.2233	0.2327	0.2468
AgCl(200)/Anl. AgCl	0.2139	0.2307	0.2408

Table S7. FWHM of 2-theta for Ag(111) diffraction.

	25% Quartile (°)	50% Quartile (°)	75% Quartile (°)
Ag(111)/NonAnl. AgBr	0.1906	0.2315	0.2678
Ag(111)/Anl. AgBr	0.2407	0.2675	0.2821
Ag(111)/NonAnl. AgCl	0.2636	0.2855	0.2990
Ag(111)/Anl. AgCl	0.2839	0.2920	0.3107

Table S8. Rotational diffusion coefficient.

	Rotational diffusion coefficient (pm^2/s)		
	ACF decay constant 25% Quartile value	ACF decay constant 50% Quartile value	ACF decay constant 75% Quartile value
AgBr(200)/NonAnl. AgBr	0.1236	0.1756	0.2535
AgBr(200)/Anl. AgBr	0.1423	0.2128	0.3621
AgCl(200)/NonAnl. AgCl	0.1194	0.1349	0.1760
AgCl(200)/Anl. AgCl	0.1202	0.1309	0.1464

Movie S1. *In situ* DXB nanoscale measurement of AgBr.

Movie S2. *In situ* DXB nanoscale measurement of AgCl.
